# A Study on Predicting the Deviation of Jet Trajectory Falling Point under the Influence of Random Wind

**DOI:** 10.3390/s24113463

**Published:** 2024-05-27

**Authors:** Hengyu Cheng, Jinsong Zhu, Sining Wang, Ke Yan, Haojie Wang

**Affiliations:** 1 School of Mechanical and Electrical Engineering, China University of Mining and Technology, Xuzhou 221006, China; tb18050003b0@cumt.edu.cn (H.C.); tb23050013a41ld@cumt.edu.cn (S.W.); ts23050139p31@cumt.edu.cn (K.Y.); ts23050202p31ty@cumt.edu.cn (H.W.); 2 China Academy of Safety Science and Technology, Beijing 100012, China

**Keywords:** random wind, jet trajectory, random forest, model prediction

## Abstract

As one of the main external factors affecting the fire extinguishing accuracy of sprinkler systems, it is necessary to analyze and study random wind. However, in practical applications, there is little research on the impact of random wind on sprinkler fire extinguishing points. To address this issue, a new random wind acquisition system was constructed in this paper, and a method for predicting jet trajectory falling points in Random Forest (RF) under the influence of random wind was proposed, and compared with the commonly used prediction model Support Vector Machine (SVM). The method in this article reduces the error in the x direction of the 50 m prediction result from 2.11 m to 1.53 m, the error in the y direction from 0.64 m to 0.6 m, and the total mean absolute error (MAE) from 31.3 to 23.5. Simultaneously, predict the falling points of jet trajectory at different distances under the influence of random wind, to demonstrate the feasibility of the proposed method in practical applications. The experimental results show that the system and method proposed in this article can effectively improve the influence of random wind on the falling points of a jet trajectory. In summary, the image acquisition system and error prediction method proposed in this article have many potential applications in fire extinguishing.

## 1. Introduction

Considering the ecological impact of fire suppression efforts [1], there has been a notable trend in the advancement of fire safety equipment towards automation and intelligence [2,3,4]. The increasing demand for automatic fire suppression systems in urban complexes underscores the push towards higher levels of automation in firefighting apparatus. The development of intelligent fire technology covers edge computing-enabled smart firefighting [5], the critical role of people in intelligent systems [6], intelligent firefighting robots [7], and robot-based intelligent firefighting from an interdisciplinary perspective [8]. Edge computing provides real-time data processing, offering firefighters faster and more accurate decision support. The involvement of individuals in intelligent systems highlights the significance of collaboration between humans and machines for the execution of complex tasks. Intelligent firefighting robots can automatically detect and extinguish fire sources, reducing personnel casualties and property losses. Furthermore, robot-based intelligent firefighting systems, combined with the changing landscape of modern infrastructure, present new challenges and future trends to promote intelligent and efficient operations.

However, the growing complexity of urban structures and the presence of hazardous chemical materials amplify the complexity and peril of fire incidents. With the advent of intelligent fire cannons equipped with various image collection devices aimed at reducing casualties and losses. Wind is one of the main factors affecting the effectiveness of fire extinguishing, and the influence of wind often complicates firefighting operations, leading to increased firefighting difficulties and extended durations of fires, thus resulting in continuous personnel casualties and property damage. Jeong et al. [9] highlighted that wind speeds ranging from 15 m/s to 22 m/s significantly increase the risk to firefighters conducting firefighting, rescue, and first aid operations under extreme wind conditions. Furthermore, Edalati-nejad et al. [10] investigated the impact of wind-driven surface fires and their effect on idealized structures on sloped terrains through numerical simulations, revealing that an increase in terrain slope leads to a rise in high-temperature areas near the building, suggesting that fire spread speed and thermal impact range also increase on steeper terrains. Consequently, firefighting and rescue operations entail heightened risks and casualties. To mitigate these challenges, the development of intelligent fire suppression systems, equipped with various image acquisition technologies, has gained traction to minimize casualties and property damage.

By analyzing and summarizing the above literature, the presence of wind during firefighting and rescue missions often exacerbates the difficulty and duration of extinguishment efforts, leading to increased risks and losses. The establishment of an accurate error prediction model can not only improve the efficiency of firefighting operations, but also reduce the harm caused by judging the effectiveness of firefighting. Therefore, it is urgent to establish a jet trajectory falling points error model under the influence of random wind.

The key contributions of this paper are summarized as follows:

(1) In response to the significant impact of current random winds on long-distance water jet flow points, this paper analyzes the advantages and disadvantages of commonly used jet trajectory acquisition methods and applies UAVs to jet trajectory collecting. Build a new random wind acquisition system that can accurately obtain real-time wind field information and water jet flow point information.

(2) Training and testing a prediction model for falling points of the jet trajectory using the RF model, and prove through the evaluation index MAE that the prediction results can be fully applied to practical fire extinguishing.

(3) Based on practical application scenarios, predict the falling points of jet trajectory at different distances, and prove that the proposed system and model can solve the problem of random wind influence in other working conditions. And compared with the prediction models under the other four kernel functions of SVM, it is proven that the prediction results of this model are better than those of the SVM prediction model.

## 2. Related Work

### 2.1. The Application of UAV Image Acquisition

In recent years, UAV-based image collection and processing methods have seen extensive applications. UAV technology has played an increasingly important role in the fire department. UAVs can quickly and efficiently obtain images and video information from the fire scene, providing valuable decision support for firefighters [11]. Deng et al. [12] proposed a UAV-based forest fire monitoring system that can monitor the spread of fires real-time and automatically send alarms. At the same time, UAVs can also be used for rescue of trapped personnel and inspection of fire hazards [13]. In urban fire protection, UAVs have also been widely applied. Ma et al. [14] used UAV images to establish a high-precision 3D urban model, providing visualization support for the fire department to formulate fire-fighting plans. Additionally, UAVs also can be utilized to monitor fire hazards in high-rise buildings, enabling early warning and guiding on-site handling [15]. Zhao [16] proposed a saliency detection algorithm for swiftly locating and segmenting core fire areas in aerial images. In addition, UAVs are widely used in many work environments due to their excellent maneuverability, such as high-altitude object recognition [17,18], agricultural inspection [19], and multi object recognition [20].

While UAVs offer broad applications, particularly in hazardous environments for image acquisition, they still lag in the realm of jet trajectory recognition and positioning. Moreover, the current methods for jet trajectory image recognition predominantly rely on binocular cameras or near-field approaches [21,22], limiting their ability to capture comprehensive fire scene information and restricting functionality to relatively close ranges. Thus, an urgent necessity exists for a novel jet trajectory acquisition method capable of providing wide viewing angles, high accuracy, reliability, and operation in adverse conditions.

Compared to commonly used image acquisition methods, UAV image acquisition methods have the following advantages in jet trajectory: (1) Flexibility and maneuverability UAV can be quickly deployed to various complex environments, including difficult to approach or dangerous places, thereby achieving real-time and continuous monitoring of jet trajectory. (2) High resolution and accuracy. UAV equipped with high-resolution cameras can capture the fine details of a jet trajectory, which is helpful for subsequent data analysis and processing; (3) Wide coverage UAV can cover large areas in a short period of time, improving the efficiency and comprehensiveness of jet trajectory acquisition. Undoubtedly, UAVs also have their drawbacks: (1) High cost. The relatively high cost of purchasing, maintaining, and operating UAVs may limit their application in large-scale or long-term monitoring projects (2) Affected by weather and environment. Adverse weather conditions or strong winds may affect the stability and image acquisition quality of UAV.

### 2.2. Random Wind Research Methods

Compared to the widespread application of UAV image acquisition, there is much less research on solving the impact of random wind, and even more so on solving the trajectory falling points of fire water jets. In existing literature, Sood Vaishali [23] introduced an autonomous robot motion control system utilizing a fuzzy logic proportional integral differential controller, thereby enabling real-time adjustments of PID gain parameters through embedded fuzzy rules. Yuan [24] proposed an N-point adaptive initialization method based on goodness of fit and statistical significance tests to compensate for fuzzy controllability between wind resistance and thrust in a ballistic dynamic model. Yao [25] developed an engineering model of wind, conducted randomization processes, and employed the Monte Carlo method for theoretical analysis to ascertain the impact of various parameters representing environmental factors on the distribution of rocket falling points. Additionally, MIHAI Lungu [26] verified the accuracy of autonomous driving considering wind shear and sensor errors via numerical simulations, while Norn Coleman [27] devised factorized implementations of extended Kalman filtering, smoothing, and prediction algorithms, discussing different wind models and validating the algorithms through simulations. In order to solve the problem of extended Kalman filtering in handling error disturbances, Zhao [28] proposed a new set-membership based hybrid Kalman filter (SM-HKF) for nonlinear state estimation under system uncertainty composed of random errors and unknown but bounded errors. A wind speed model describing the relationship between airship speed and wind speed was established using the information output from ADS, and a mathematical model for SINS/GNSS/ADS integrated navigation was further established. Based on these models, a constrained UPF was developed to obtain system state estimation for SINS/GNSS/ADS integration. The proposed constraint UPF uses a wind speed model to constrain the UPF filtering process, effectively resisting the influence of the wind field on navigation solutions [29]. From the above literature, it can be seen that most of the error compensation for random wind is mainly achieved by establishing a random wind field model or designing a controller to suppress the impact of random wind.

## 3. Jet Trajectory Model Prediction

RF and SVM perform well in handling complex data and pattern recognition. The prediction of the landing point of a jet trajectory under the influence of random wind involves multiple variables and complex nonlinear relationships, and both algorithms can effectively handle this complexity. RF improves prediction accuracy by integrating multiple decision trees, while automatically processing feature selection and importance assessment. SVM achieves classification or regression by searching for the maximum interval hyperplane in high-dimensional space, which is very effective for handling high-dimensional data and complex nonlinear relationships.

Although there are other methods that can be used for prediction tasks, such as neural networks, deep learning, etc., they may require more data and computational resources when dealing with such problems, and may be more prone to overfitting or unstable training. In contrast, RF and SVM typically have advantages in balancing predictive performance, computational efficiency, and stability. And these characteristics happen to be the most needed for firefighting and rescue.

### 3.1. Random Forest Model

The Random Forest (RF) is a powerful ensemble learning method designed for both classification and regression tasks, effectively constructing multiple decision trees during training. It outputs the mode of classes for classification or the mean prediction for regression, thereby enhancing accuracy and reducing the risk of overfitting associated with individual decision trees [30]. This method excels in handling high-dimensional spaces and maintains accuracy even with a significant amount of missing data. Random Forests harness the power of ensemble learning by creating a ‘forest’ of decision trees, with each tree built on a randomly selected subset of training data and variables. This randomness ensures diversity among the trees, which is crucial for combating overfitting. Additionally, Random Forests are renowned for their ability to rank the importance of variables in classification, providing valuable insights for predictive tasks. In conclusion, the Random Forest model emerges as a reliable choice for a wide range of applications, attributed to its adaptability to different data types, robustness to high-dimensional data and multicollinearity, and reduced risk of overfitting [31].

**Figure 1:**
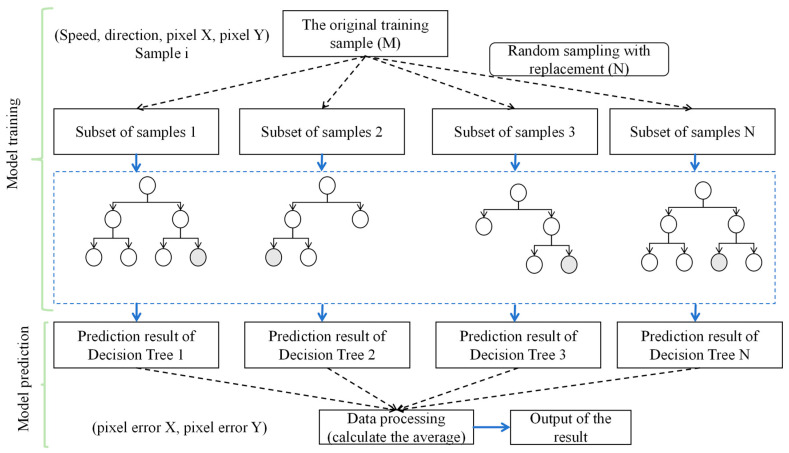
RF prediction falling points flowchart.

Accurate prediction of the jet trajectory is crucial for various applications, and data-driven modeling techniques like random forests have emerged as promising alternatives to traditional computational fluid dynamics (CFD) simulations or empirical correlations [32,33]. Several studies have explored the application of random forest models for jet trajectory prediction, demonstrating good accuracy and computational efficiency compared to CFD and traditional methods [34]. These models have shown the ability to capture complex jet dynamics, including the effects of nozzle geometry, operating conditions, and swirl intensity [35]. The integration of random forest models with jet trajectory prediction is a promising approach, offering advantages in terms of computational efficiency and adaptability to various jet configurations. The RF model prediction process is shown in Figure 1.

In the realm of machine learning, the random forest algorithm emerges as an ensemble approach for datasets *D* encompassing m samples and n features. Initially, this model adopts bootstrap sampling to allocate *m* samples to each tree, a methodology permitting repetition in sample selection, thereby allowing the same sample to be chosen multiple times, while certain samples may not be chosen at all. Subsequently, at every decision node, instead of scrutinizing all *n* features to determine the data division, the model randomly selects a subset of features. Decisions regarding the optimal split rely solely on this subset, which is significantly smaller than *n*, aiming to reduce the correlation among trees and thereby enhance the generalization ability of the model. This process repeats until each tree attains the pre-established maximum depth or the sample count at node declines below a specified threshold. It is noteworthy that, unlike certain other models, the decision trees in this context undergo no pruning. Ultimately, upon the construction of all trees, the random forest consolidates the predictive outcomes from every tree to render a final verdict: for regression tasks, this typically entails computing the mean of all trees’ predictions; for classification scenarios, a majority vote is employed. 

Through this methodology, the random forest endeavors to encapsulate the complex structure within *D*, simultaneously mitigating the risk of overfitting by integrating randomness, thereby enhancing the efficacy of model on unseen data. Owing to its simplicity in construction, intelligibility, adaptability, and proven empirical success, random forests are extensively applied across diverse settings.

### 3.2. Support Vector Machine Model Prediction (SVM)

In addition to random forests, support vector machines are also a powerful data-driven modeling approach, widely applied in classification and regression problems [36] The basic idea of SVMs is to find an optimal hyperplane that maximizes the margin between different classes of data, thereby achieving effective classification [37,38]. This optimal hyperplane is determined by only a few critical training samples, making SVMs highly robust to high-dimensional data and noisy data [39].

Several studies have explored the application of SVMs for jet trajectory prediction [40]. Compared to traditional methods, SVM models have demonstrated higher prediction accuracy and computational efficiency, while also being able to capture the complex jet dynamics, such as the effects of nozzle shape and operating conditions. This combination of data-driven models and physical insights provides a reliable and efficient solution for jet trajectory prediction.

In the prediction phase, once the SVM model learns this optimal hyperplane from the training data, it can be used to classify new, unseen data. For a new data point, the SVM projects it onto the learned hyperplane and predicts its class based on which side of the hyperplane it falls on. If a regression problem is considered, the predicted value will be the distance from the hyperplane to the data points. 

During the training phase, the SVM model learns how to predict target values based on input feature vectors. This process involves selecting an optimal hyperplane to separate samples of different categories. The hyperplane equation is:

(1) wx+b=0

Among them, *w* is the weight vector, *x* is the input feature vector, and *b* is the bias term. This hyperplane is used to partition samples of different categories.

The training process of SVM can be transformed into a quadratic programming problem, with the goal of minimizing the following function:

(2) $$\left[\frac{1}{2}{\left\|w\right\|}^{2}+C\sum_{1}^{n}\xi_{i}\right]$$

Among them, *C* is the regularization parameter used to control the punishment level of the error term, *ξ_i_* is the relaxation variable used to handle indivisible samples, and n is the number of samples.

In the prediction phase, SVM uses the hyperplane learned during the training phase to classify and regress new data, calculates the given new data points, and uses the following formula to calculate the decision function:

(3) f=wx+b

Determine the prediction category of new data points based on the sign of the decision function *f*(*x*). Usually, the sign function sign is used to determine the predicted category. If *f*(*x*) ≥ 0, the sample belongs to positive class; if *f*(*x*) < 0, the sample belongs to the negative class.

(4) f(x)=sign(wx+b)

In this article, we select four commonly used kernel functions for SVM training and prediction, and the kernel function formula is as follows:

Linear kernel function:

(5) $$K(x_{i},y_{i})={x_{i}}^{T}x_{j}$$

Polynomial kernel function:

(6) $$K(x_{i},y_{i})={\left(\gamma {x_{i}}^{T}x_{j}+r\right)}^{d},>0$$

Sigmoid kernel function:

(7) $$K(x_{i},y_{i})=tanh(\;\gamma {x_{i}}^{T}x_{j}+r)$$

Radial basis kernel function:

(8) $$K(x_{i},y_{i})=exp{\left(-\gamma \left\|x_{i}-x_{j}\right\|\;\right)}^{2},\gamma >0$$

In order to evaluate the effectiveness of model predictions, the mean absolute error (MAE) is usually used as the basis for evaluating the quality of the model. Its definition is as follows:

(9) $$MAE=\frac{1}{n}\sum_{i=1}^{n}\left|x_{i}-{\hat{x}}_{i}\right|$$

where *n* is the number of samples, *x_i_* is the actual value of the *i*-th sample, and ${\hat{x}}_{i}$ is the predicted value of the *i*-th sample.

This article used RF prediction model to predict the falling points of jet trajectory under the influence of random winds, and compared the prediction accuracy of SVM models to determine that RF has better prediction accuracy in combating the influence of random winds on jet trajectory. It has been proven that the use of RF is superior in predicting jet trajectory landing points in UAV images. It played a very important role in improving the efficiency of fire extinguishing.

## 4. Experiment Establishment

### 4.1. UAV Layout Method

**Figure 2:**
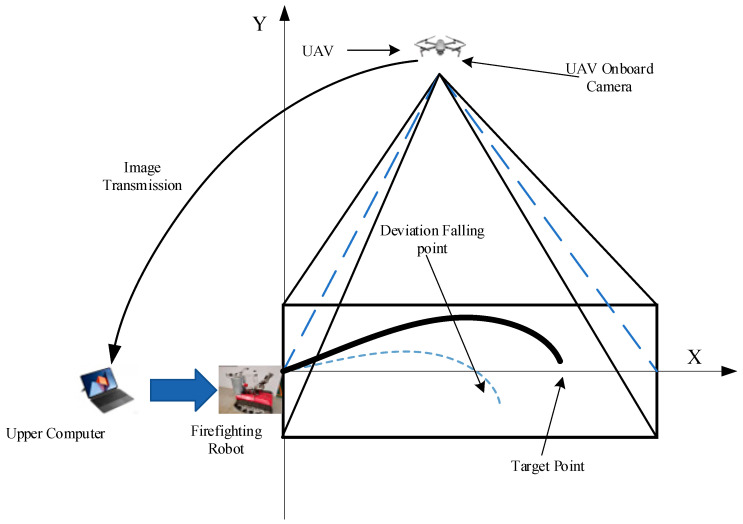
Schematic diagram of jet trajectory image acquisition under UAV.

This experimental study was undertaken at Ling Tian Co., Ltd. in Xuzhou, Jiangsu, China, a location characterized by its expansive area and the absence of surrounding obstructions. Such advantages are deemed crucial for the collection of jet trajectory imagery and wind-related data. Given the prohibitions on ignition within the confines of the test site, alternative methodologies were employed. Specifically, a UAV was elevated to an optimal altitude, and its onboard camera system was meticulously adjusted to achieve a 90-degree downward orientation. Airborne camera capture images with a resolution of 1200 × 932 pixels. This setup facilitated the comprehensive capture of the jet trajectory imagery, crucial for the subsequent processing and analysis. The schematic diagram of the UAV obtaining jet trajectory falling points and target point position information is depicted in Figure 2.

### 4.2. Wind Speed and Direction Measurement Collection

**Figure 3:**
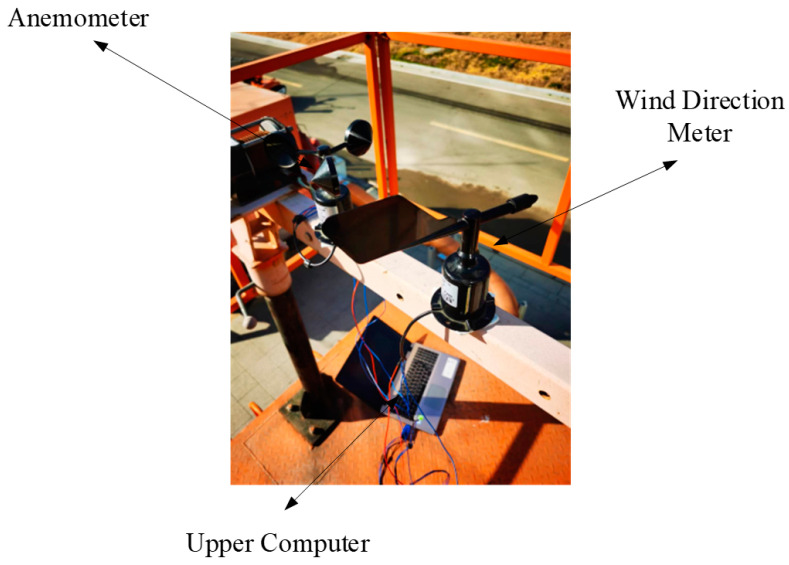
Random wind information collection system.

The selection of the location for the installation of wind speed and direction measurement instruments must adhere to meteorological standards to guarantee the representativeness of the collected wind data, and it is imperative to circumvent any obstruction effects posed by terrain and buildings. An area characterized by open and flat terrain, devoid of obstacles, is optimal to ensure the precise measurement of wind speed and direction by the anemometer and wind vane. Consideration must be given to variations in meteorological conditions, such as temperature, humidity, atmospheric pressure, and other environmental factors, which may influence the accuracy of measurements. Owing to site constraints, the instruments for measuring wind speed and direction are positioned approximately 4 m above the ground level, marking the highest point in the immediate vicinity, thus eliminating nearby obstructions to enhance the reliability of wind data collection. As shown in Figure 3. The orientation for wind speed and direction measurement is standardized to true north, with true north designated as 0°. The parameters of the random wind collection equipment are shown in Table 1.

### 4.3. Acquisition of Jet Trajectory Falling Points Error Data Based on UAV Images

**Figure 4:**
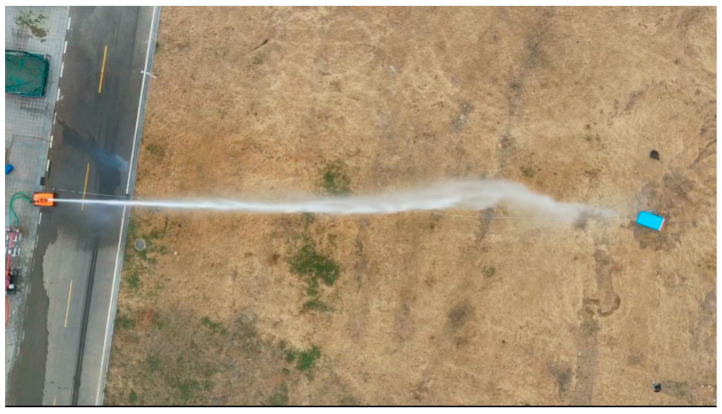
Images obtained from UAV camera.

In accordance with the specifications set forth by the wind anemometer, with the orientation of true north established at 0°, the initial direction of the fire monitor is aligned to true north, and a target point is designated 50 m due north. The protocol mandates that the jet be discharged towards the target when the wind speed approximates 0 m/s. Upon the jet impact at the target location, measurements of wind speed and direction commence, accompanied by the acquisition of imagery capturing deviations in the jet trajectory. Furthermore, the instantaneous wind speed and direction at the moment of trajectory error are meticulously documented. Table 2 depicts the pixel position information related to the falling points error of 20 jet trajectory affected by random wind. It is defined within the parameters of this study that a positive x-direction indicates the jet trajectory falling points has not reached the predetermined target, whereas a positive y-direction signifies the jet trajectory falling points is positioned above the y-axis. The image obtained from the perspective of the UAV is shown in Figure 4.

### 4.4. Experimental Steps and Parameter Settings

This article collects 200 data samples at each distance, each containing wind speed, direction, and the X-direction deviation of the corresponding time point pixel, as well as the Y-direction deviation of the corresponding time point pixel. In order to collect the pixel bias dataset, this article uses UAV to collect image data, and provides the pixel bias of each sample through image and manual processing methods. During the model training process, the data is divided into training and testing sets, with a ratio of 7:3. Random Forest randomly selects *K* samples from *M* training sample sets that have been put back as a subset of samples. Assuming there are *N* decisions, we obtain a total of *N* sample subsets. At this point, the data in each sample subset is different, which makes the features learned by each decision tree different, increasing the richness of the data. Then, each decision tree undergoes regression learning based on sample features, and the final decision tree is obtained through model training. When the model is trained well, we give a new wind speed and direction value, and multiple decision trees calculate their respective pixel deviations. Here, we represent the final result by calculating the average value of different decision trees, which alleviates the impact of incorrect predictions caused by a single decision tree not learning. Set the minimum number of samples required for dividing the internal points of the random forest to 2, and the minimum number of samples required for leaf nodes to 1, choose MSE as the metric for the quality of bright splitting. Set the number of features to consider when searching for the optimal splitting point to ‘auto’. All others are default values.

When using SVM for model training, to prevent overfitting, the regularization parameter C is set to 1.0, and the tolerance of the loss function is set to 0.1. The order of the polynomial kernel function is set to 3.

## 5. Experimental Results and Analysis

### 5.1. Experimental Device

The experimental setup primarily consists of a fire suppression system, including firefighting robots. The platform is further augmented with additional equipment such as UAV equipped with aerial cameras, fire cannons. Image data captured by the UAV is transmitted to a central processing unit via wireless communication technologies, where image analysis is performed. A target location is established 50 m directly north of the fire monitor position. A proprietary software application facilitates the modification of the fire monitor azimuth and elevation angles through a control interface.

**Figure 5:**
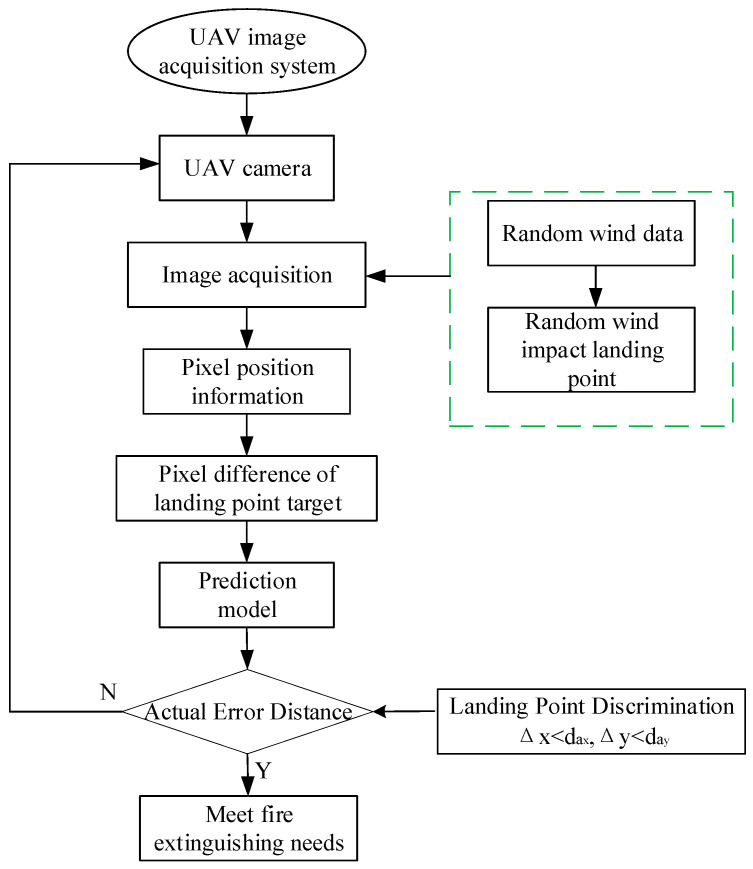
Model prediction establishment flowchart.

To elucidate the procedural sequence and recent advancements in the method for image acquisition and the prediction of jet trajectory falling points, a comprehensive workflow is depicted in Figure 5. This illustration serves to provide a visual representation of the operational framework and the progress achieved in the implementation of the proposed methodology. Firstly, obtain the jet trajectory image and random wind information at the same time through UAV. Then train the collected data. Finally, we test the trained model. To verify the predictive performance of the model, we use GSD to convert the average absolute error into actual distance error and compare it with the actual measured coverage in Table 3 to determine whether the predicted results of the model meet the actual firefighting needs.

The pixel difference is converted to the actual distance *d_a_* using the Ground Sampling Distance (GSD) [41] in this article, GSD is a parameter that describes the ground size corresponding to a single pixel in a digital image, representing the distance between the center points of two consecutive pixels, by calculating GSD, we can understand the actual ground size represented by each pixel in the image captured by the UAV, which is crucial for flight planning, data acquisition, and subsequent data processing. and the calculation formula is as follows:

(10) $$d_{a}=GSD*d_{p}=\frac{HS_{s}}{fS_{p}}d_{p}$$

Among them, *d_p_* represents pixel deviation, *H* represents UAV flight altitude, *S_s_* represents sensor size, and *S_p_* represents image size.

Measurement of jet trajectory coverage at different distances:

Measure the coverage of the jet trajectory at different distances when there is no wind, and obtain the water jet coverage data at 30–60 m through actual measurement. As shown in Table 3.

In order to better determine the predictive performance of the method proposed in this paper, even if the jet trajectory in the x direction is affected by factors such as initial velocity, random wind, and air resistance, resulting in a longer coverage distance in the x direction, we choose the y direction coverage distance as the farthest coverage distance. For example, at 50 m, when the coverage distance in the x direction is less than 2.5 m and the coverage distance in the y direction is also less than 2.5 m, it can be considered that the prediction model meets the fire extinguishing requirements.

### 5.2. Analysis of Experimental Results

**Figure 6:**
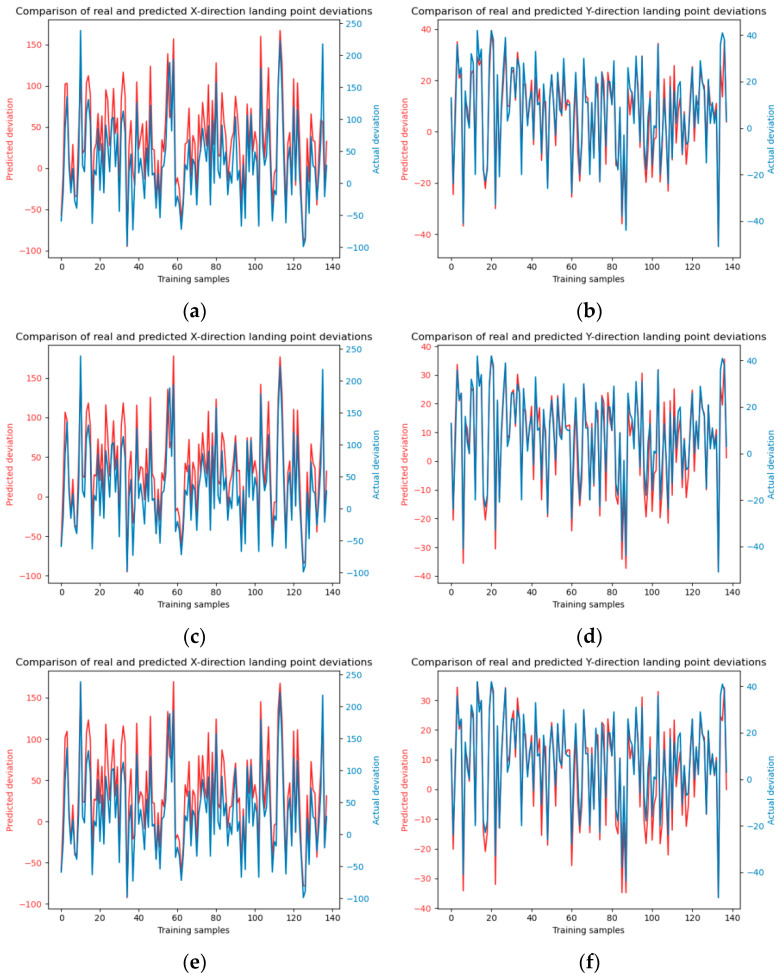
Illustrates the training process of the Random Forest model. Panels (**a**) and (**b**) depict the model training in the x and y directions, respectively. The number of decision trees is 10. Panels (**c**) and (**d**) show the model training in the x and y directions with decision trees number of 20, panels (**e**) and (**f**) present the model training in the x and y directions using decision trees number of 30.

**Figure 7:**
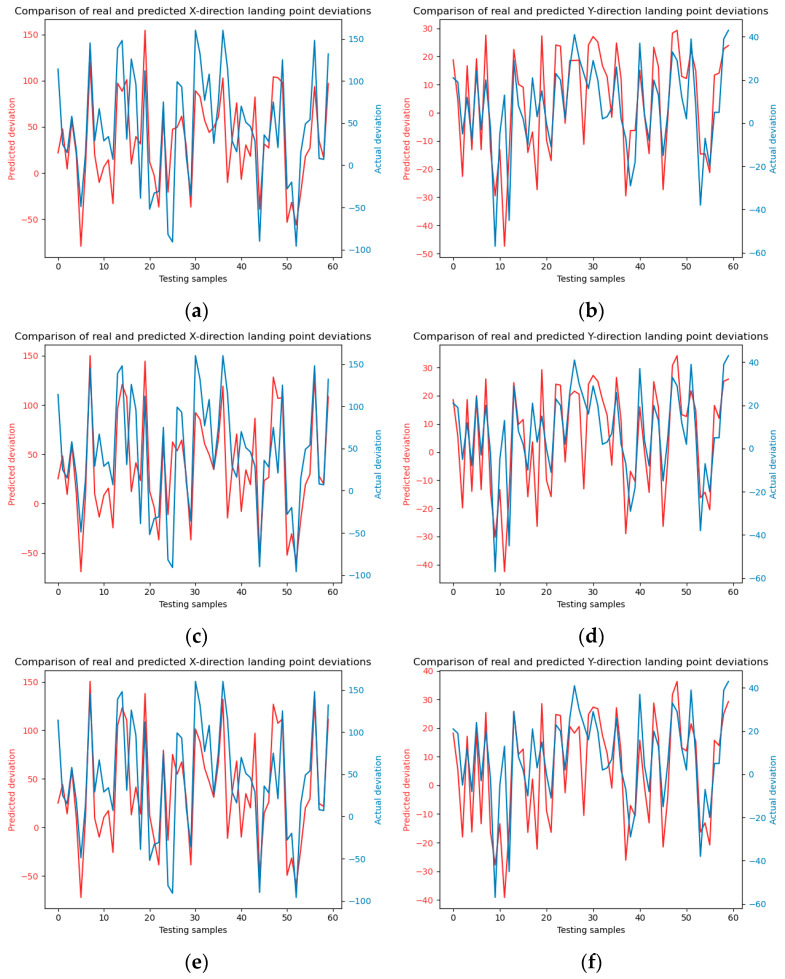
Showcases the testing phase of the Random Forest model, following the training process described in Figure 5. Panels (**a**) and (**b**) display the model predictions in the x and y directions, respectively, using a decision trees number of 10. Panels (**c**) and (**d**) illustrate the model predictions in the x and y directions with a decision trees number of 20. Panels (**e**) and (**f**) depict the predictions in the x and y directions using a decision trees number of 30.

To augment the comparative analysis of our experimental outcomes with alternative approaches, the dataset was partitioned into a training set and a test set with a ratio of 7:3. Subsequently, the Random Forest and SVM (Support Vector Machine) methodologies were employed to forecast the falling points of jet trajectory. The model training and testing results are shown in Figure 6 and Figure 7.

**Figure 8:**
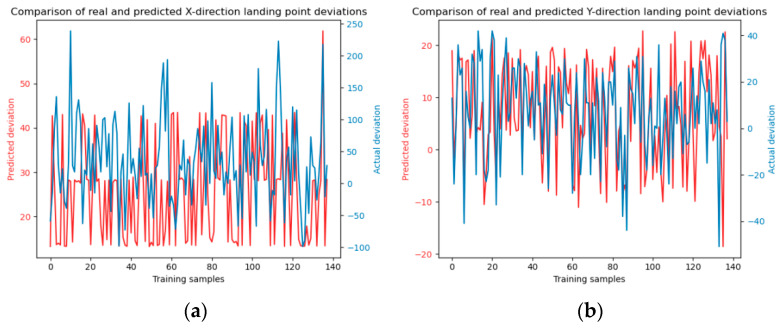
Linear kernel function model training. Panels (**a**) and (**b**) depict the model training in the x and y directions, respectively.

**Figure 9:**
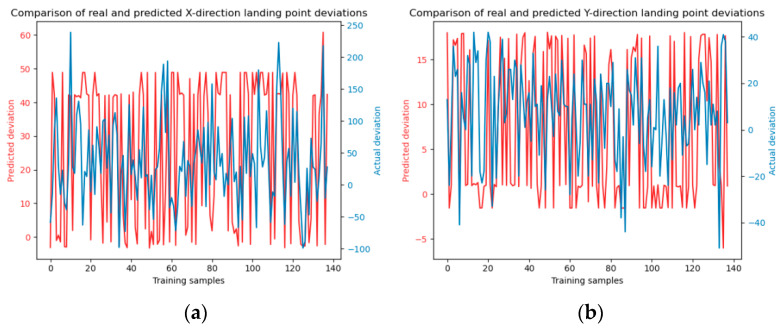
Polynomial kernel function model training. Panels (**a**) and (**b**) depict the model training in the x and y directions, respectively.

**Figure 10:**
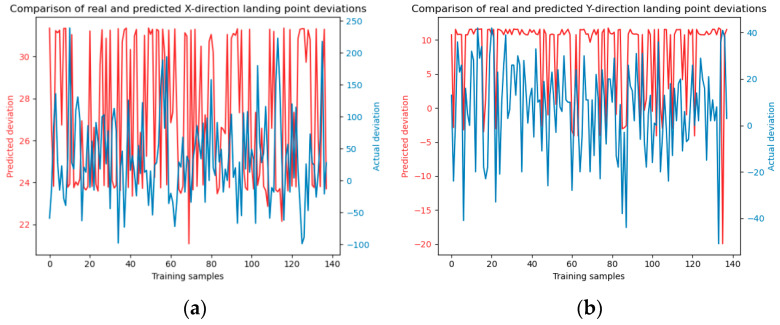
Sigmoid kernel function model training. Panels (**a**) and (**b**) depict the model training in the x and y directions, respectively.

**Figure 11:**
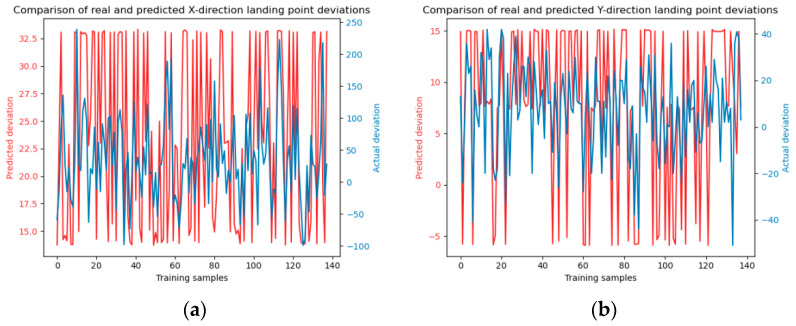
Radial basis kernel function model training. Panels (**a**) and (**b**) depict the model training in the x and y directions, respectively.

The results of SVM model training using four kernel functions are shown in Figure 8, Figure 9, Figure 10 and Figure 11.

**Figure 12:**
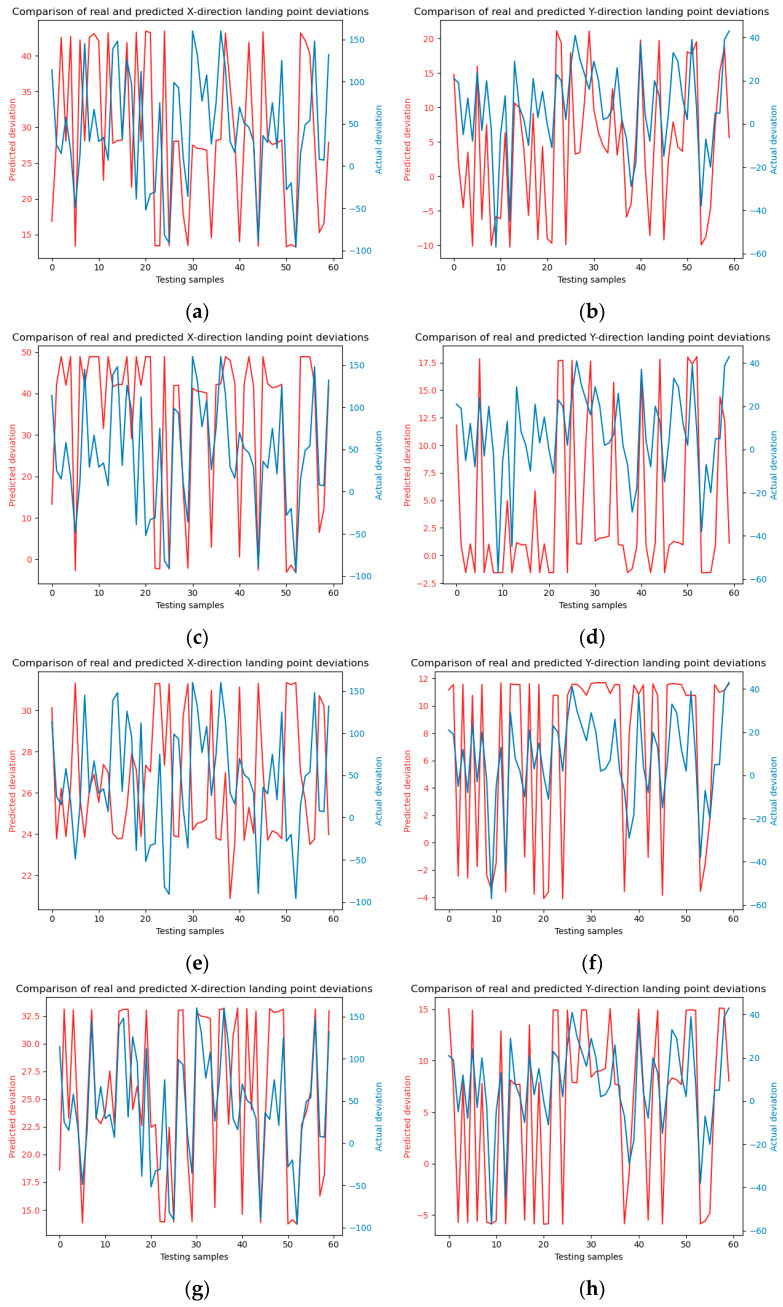
SVM model prediction. (**a**) and (**b**) are linear kernel functions; (**c**) and (**d**) are polynomial kernel functions; (**e**) and (**f**) are Sigmoid kernel functions; (**g**) and (**h**) are radial basis kernel functions.

The SVM model test results under four kernel functions are shown in Figure 12.

The MAE results generated after model training and testing are shown in Table 4, Table 5, Table 6 and Table 7.

As shown in Table 5. The SVM model, across its four kernel functions training sets, yields a mean absolute error (MAE) of approximately 30. This figure significantly exceeds the random forest model MAE, which is around 9 in Table 4. indicating that the random forest model demonstrates superior accuracy in predicting jet trajectory falling points. Specifically, the random forest model exhibits a more adept capability in addressing the prediction of jet trajectory falling points, making it the preferable choice for such applications. Although the MAE values obtained after model testing in Table 6 are relatively large. But the proportional calculations results reveal that for random forests consisting of 10, 20, and 30 trees, the observed deviations in the x-direction are 1.7 m, 1.57 m, and 1.53 m, respectively, while in the y-direction, they are 0.63 m, 0.61 m, and 0.6 m, respectively. These deviations fall within acceptable ranges for practical firefighting operations. In contrast, by calculating the proportion of MAE in Table 7, it can be concluded that deviations associated with the four kernel functions SVM model in the x-direction are 2.28 m, 2.11 m, 2.38 m, and 2.11 m, and in the y-direction, they are 0.66 m, 0.77 m, 0.68 m, and 0.64 m. This highlights the superior predictive efficacy of the random forest model compared to the SVM model for this application. This proves that both RF and SVM prediction results meet the fire extinguishing requirements, but the RF model has smaller prediction errors and higher accuracy.

By comparing Table 4 and Table 6, it can be seen that the training results of the RF model are much better than those of SVM. When the number of decision trees is 30, the total MAE is only 9.0, which is much smaller than the total MAE of SVM. This indicates that the prediction of jet trajectory falling points under the influence of random wind has better accuracy. Comparing the MAE in the x and y directions, it can be seen that the prediction error of the falling points in the y direction is only 4 pixels, and the prediction error in the x direction is also smaller than that of the SVM model.

Moreover, a comparison of predicted falling points in the x and y directions reveals a more accurate prediction in the y-direction. This discrepancy can be attributed to the initial conditions of jet trajectory, where the initial velocity in the x-direction is present and the velocity component in the y-direction is approximately zero. Consequently, changes in velocity in the x-direction are influenced by both the previous velocity and the component of wind in the x-axis direction. Conversely, in the y-direction, the trajectory is primarily affected by wind speed, with negligible impact from the initial velocity.

### 5.3. Comparison of Prediction Methods at Different Distances

**Figure 13:**
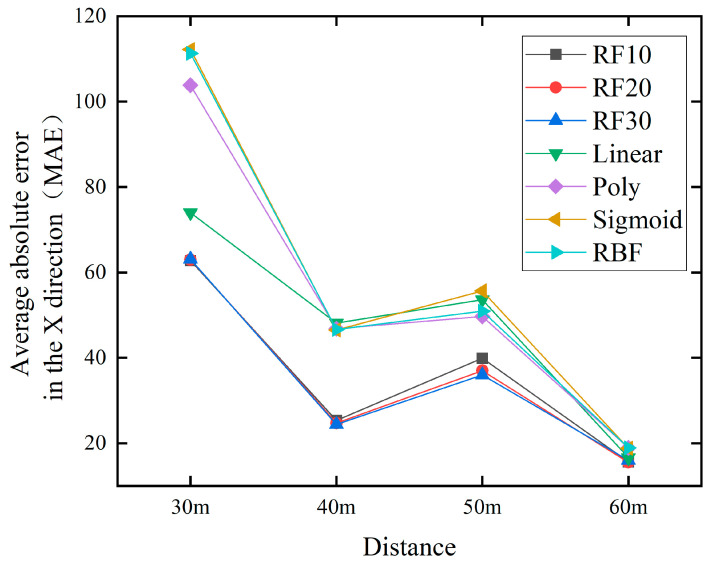
Comparison chart of jet trajectory falling points prediction at different distances in the x direction.

**Figure 14:**
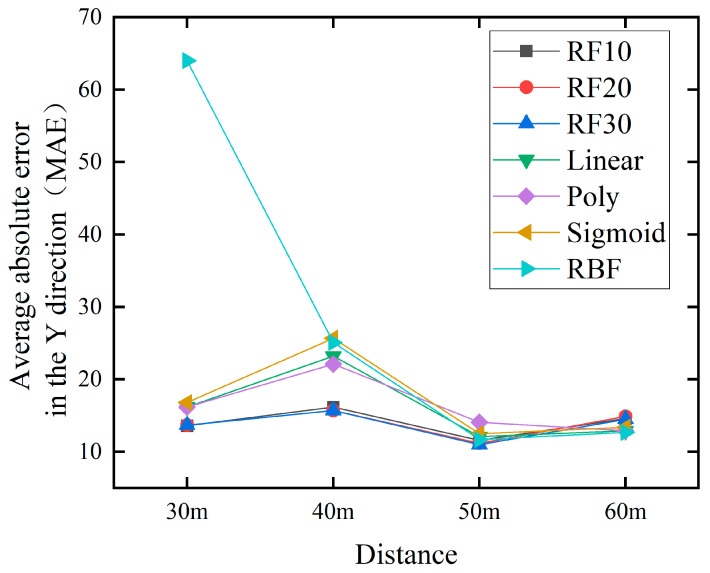
Comparison chart of jet trajectory falling point prediction at different distances in the x direction.

In order to better validate the effectiveness and performance of the method proposed in this paper, we applied it to predict water jet falling points at distances of 30 m, 40 m, and 60 m. And compare the methods proposed in the previous text, as shown in Figure 13 and Figure 14.

Figure 13 and Figure 14 depict the MAE of RF and SVM at different distances, respectively. From the image, we will compare the overall best RF30 with the best SVM prediction results. In the x direction, at 30 m, the values are 63.1 and 74.0, respectively. At 40 m, it is 24.4 and 46.5 respectively. At 50 m, the values are 36.0 and 49.7 respectively. At 60 m, it is 16.0 and 16.6 respectively. In the y-direction, at 30 m, the values are 13.7 and 16.2 respectively. At 40 m, it is 15.7 and 22.1 respectively. At 50 m, the values are 11.0 and 11.7, respectively. At 60 m, it is 14.5 and 12.7 respectively.

By analyzing two prediction models, the main reasons why RF performs better than SVM are as follows:

1. RF is an ensemble learning model composed of multiple decision trees, which improves overall prediction performance by combining the prediction results of multiple weak learners. For complex datasets, RF can capture more features and non-linear relationships between them, making them more suitable for predicting the falling points of jet trajectory. The goal of SVM is to find an optimal hyperplane and separate data of different categories. For some complex or highly nonlinear datasets, SVM may find it difficult to find a suitable hyperplane, leading to a decrease in prediction performance.

2. RF can alleviate the impact of noise and outliers on prediction results to a certain extent by integrating multiple decision trees. Each decision tree randomly selects features and split points during the construction process, which helps to reduce the impact of outliers on overall prediction. SVM is sensitive to noise and outliers as they may significantly affect the position of the hyperplane. When there is a lot of noise or outliers in the dataset, the performance of SVM may be greatly affected.

3. RF can provide feature importance assessment, which helps identify features that have a significant impact on the results during the prediction process. For the prediction of jet trajectory falling points, which requires considering multiple physical factors, feature selection is particularly important. SVM is relatively weak in feature selection and mainly relies on kernel functions to handle nonlinear relationships, unlike RF that can directly evaluate the importance of each feature.

Compared with the SVM prediction model, RF has smaller model prediction bias and higher prediction accuracy, especially in the x-direction when factors such as initial jet velocity and air resistance are superimposed. RF has better performance in model prediction results. And the prediction accuracy of the model also improves with the increase of the number of decision trees.

The minimum MAE of the prediction results for 30 m, 40 m, 50 m, and 60 m were calculated and displayed in Table 8. It can be seen that the RF model has better prediction performance than the SVM model at all four distances.

## 6. Conclusions

A fire water jet falling points acquisition system affected by random wind has been established, which can timely obtain jet trajectory falling points and fire scene image information and real time obtain random wind speed and direction information. Train and test the RF prediction model based on the obtained information. By comparing with the SVM prediction model, the superiority of this model in predicting falling points has been verified. Taking 50 m as an example, the minimum error from calculation to prediction is 1.53 m and 0.6 m, which fully meets the fire extinguishing needs. This method can improve the prediction performance of falling points. From the total MAE tested, it can be seen that the method proposed in this article can reduce the deviation by at least 7 pixels compared to SVM.

In addition, to verify the feasibility of the proposed method in practical applications, predictions were made for the falling points at different distances, and model evaluation indicators MAE were used to prove that the proposed method is superior to the SVM prediction model at each distance. At the same time, it also proves that the system and method proposed in this article can play a good predictive role in long-distance (50 m, 60 m) fire extinguishing.

Our future work mainly includes two aspects. 1. Further improve the prediction accuracy of jet falling points. There are many interference factors that can cause deviation in the falling points, such as UAV drift and shaking, fluctuations in supply pressure, and air resistance. 2. Collection system and prediction model should be further extended.

## Figures and Tables

**Table 1:** Random wind collection equipment parameters.

Parameter	Anemometer	Wind Direction Meter
Type	RS-FXJT-N01-360	RS-FSJT-N01
Power supply	DC 10–30 V	10–30 V
Output	RS485	RS485
Measuring range	0–30 m/s	0–360°
Resolution ratio	0.1 m/s	/
Dynamic response time	≤0.5 s	≤0.5 s
Start wind speed	≤0.2 s	/

**Table 2:** Jet trajectory pixel error.

Serial Number	Wind Speed	Wind Direction	X Direction Pixel Error	Y Direction Pixel Error
1	0.4	173.6°	39	−5
2	0.9	175.7°	91	−18
3	1	178.1°	62	−21
4	1.2	179.6°	29	−20
5	1.8	3.2°	115	−7
6	1.4	14.6°	14	−3
7	1.1	344.7°	38	11
8	0.9	181.8°	31	2
9	0.4	1.1°	−34	−8
10	2.7	189. 4°	160	29
11	3.2	183.6°	194	10
12	1.6	341.2°	104	15
13	1.4	316.8°	114	21
14	1.1	182.3°	85	2
15	0.7	316.6°	82	11
16	1.7	34.6°	18	−18
17	1.5	45.4°	28	−24
18	1.8	27.9°	60	−20
19	2.3	348.1°	15	23
20	319.8	7°	39	13

**Table 3:** Coverage distance of water jet flow points at different distances without the influence of random wind.

Distance	Flight Altitude	x-Direction	y-Direction
30 m	26 m	7 m	2 m
40 m	39 m	8.5 m	2.2 m
50 m	51 m	10.2 m	2.5 m
60 m	60 m	12 m	4 m

**Table 4:** MAE for RF model training.

Number of Decision Trees	X-MAE	Y-MAE	Total-MAE
10	16.0	4.2	10.1
20	14.7	4.0	9.4
30	14.0	4.0	9.0

**Table 5:** MAE for SVM model training.

Kernel Function	X-MAE	Y-MAE	Total-MAE
Linear kernel function	50.9	12.4	31.1
Polynomial kernel function	47.0	13.6	30.3
Sigmoid kernel function	52.6	13.4	33.0
Radial basis kernel function	46.9	12.3	29.6

**Table 6:** MAE for RF model testing.

Number of Decision Trees	X-MAE	Y-MAE	Total-MAE
10	39.9	11.6	25.7
20	37.0	11.2	24.1
30	36.0	11.0	23.5

**Table 7:** MAE for SVM model testing.

Kernel Function	X-MAE	Y-MAE	Total-MAE
Linear kernel function	53.6	12.1	32.9
Polynomial kernel function	49.7	14.1	31.9
Sigmoid kernel function	55.7	12.5	34.1
Radial basis kernel function	50.9	11.7	31.3

**Table 8:** Minimum Total MAE at 30 m, 40 m, 50 m, and 60 m.

Distance	RF	SVM
30 m	38.1	45.1
40 m	20.7	34.5
50 m	23.5	31.3
60 m	15.2	15.8

## Data Availability

Data are contained within the article.
